# Early Impact of Mobilization Process on Cardiac Function and Size in Patients Undergoing Autologous Hematopoietic Stem Cell Transplantation

**DOI:** 10.3390/jcm13030773

**Published:** 2024-01-29

**Authors:** Audrone Vaitiekiene, Migle Kulboke, Monika Bieseviciene, Agne Bartnykaite, Benas Kireilis, Diana Rinkuniene, Antanas Jankauskas, Justinas Zemaitis, Ignas Gaidamavicius, Rolandas Gerbutavicius, Domas Vaitiekus, Jolanta Justina Vaskelyte, Gintare Sakalyte

**Affiliations:** 1 Department of Cardiology, Medical Academy, Lithuanian University of Health Sciences, 44307 Kaunas, Lithuaniajolanta.vaskelyte@lsmu.lt (J.J.V.);; 2 Department of Oncology and Hematology, Medical Academy, Lithuanian University of Health Sciences, 44307 Kaunas, Lithuania; 3 Oncology Research Laboratory, Oncology Institute, Lithuanian University of Health Sciences, 50161 Kaunas, Lithuania; 4 Medical Academy, Lithuanian University of Health Sciences, 44307 Kaunas, Lithuania; 5 Institute of Physiology and Pharmacology, Medical Academy, Lithuanian University of Health Sciences, 44307 Kaunas, Lithuania; 6 Department of Radiology, Medical Academy, Lithuanian University of Health Sciences, 44307 Kaunas, Lithuania; 7 Institute of Cardiology, Lithuanian University of Health Sciences, 47181 Kaunas, Lithuania

**Keywords:** autologous hematopoietic stem cell transplantation, cardiotoxicity, mobilization

## Abstract

**Background:** The hematopoietic stem cell transplantation (HSCT) process is known to cause cardiac toxicity of different grades. In this paper, we aimed to evaluate the impact of mobilization procedure of hematopoietic stem cells for autologous HSCT process for left and right ventricle sizes and functions. **Material and Methods:** The data of 47 patients undergoing autologous HSCT were analyzed. All patients underwent hematopoietic stem cell mobilization with chemotherapy and filgrastim at 10 µg/kg/d. Echocardiography was performed two times: before enrolling in the transplantation process and after mobilization before the conditioning regimen for transplantation. Changes in left and right ventricle (RV) diameter and systolic and diastolic function of the left ventricle and systolic function of the RV were measured. **Results**: A statistically significant difference was observed in the change of right ventricular function (S‘)—it slightly decreased. Mean S‘ before mobilization was 13.93 ± 2.85 cm/s, and after mobilization it was 12.19 ± 2.64 cm/s (*p* = 0.003). No statistically significant change in left ventricular diameter and systolic and diastolic function and RV diameter was observed. **Conclusions**: The mobilization procedure in patients undergoing autologous HSCT is associated with reduced RV systolic function. S‘ could be used as a reliable tool to evaluate early cardiotoxicity in HSCT patients and guide further follow-up.

## 1. Introduction

Hematopoietic stem cell transplantation (HSCT) is a potentially curative procedure for various malignant hematologic and lymphoid diseases and some solid tumors [1,2]. Two fundamentally different types of HSCT are categorized by the source of the stem cells. Autologous HSCT represents infusion of the patient’s own hematopoietic stem cells (HSCs), and allogeneic HSCT refers to the infusion of HSCs obtained from a donor via bone marrow harvest or apheresis [3].

Autologous HSCT provides hematopoietic support after high-dose chemotherapy and is used for treatment of patients with multiple myeloma (MM) or some types of chemosensitive relapsed lymphomas and a few solid tumors [4]. Hematopoietic cells (HCs) and progenitor cells are released from the bone marrow into the peripheral blood through a process called mobilization. HSCs are then collected from the blood—the procedure is called apheresis—and cryopreserved for administration after treating the main disease with the high-dose preparative regimen (conditioning) [1].

According to the Worldwide Network of Blood and Marrow Transplantation (WBMT), the global count of HSCTs has been consistently rising by approximately 7% annually, reaching an average of around 90,000 per year. By 2019, a total of one-and-a-half million patients had undergone HSCT worldwide since 1957 [5]. There is a high likelihood that post-HSCT patients that have surpassed five years without relapse have a high probability of surviving for an additional 15 years [6]. Even though HSCT has reached much higher survival rates and is significantly improved compared to the past, this procedure is still related with some severe acute or chronic complications. Apart from the main complications, which are graft-versus-host disease or various infections, the HSCT process is also known to cause cardiac toxicity of different grades, which present either short-term or long-term complications [7].

In the course of HSCT, as well as within the initial 100 days following the HSCT, various cardiovascular events might occur, including acute heart failure, life-threatening arrhythmias, pericardial tamponade, and even cardiac arrest. Also, various long-term cardiovascular complications can arise, such as valvular dysfunction, cardiomyopathy, and ischemic heart disease [8]. There are numerous predisposing conditions that may worsen cardiac outcomes, for instance, diabetes mellitus, hypertension, age and, most importantly, previous treatment with chemotherapy [9]. Recently published guidelines of cardio-oncology developed by the European Society of Cardiology (ESC) classified cardiotoxicity risk according to the type of HSCT (allogeneic versus autologous), cardiovascular risk factors, preexisting cardiovascular morbidities, and previous cardiotoxic anticancer treatment effects [10].

In order to reduce the occurrence and intensity of cardiac complications, the guidelines mentioned above advise to conduct assessment of cardiac function before undergoing HSCT. One of the most used and currently available imaging methods is echocardiography, which is recommended for evaluation of left ventricular ejection fraction (LV EF). Two-dimensional echocardiography is the first-choice method for detecting cardiotoxic effects, as it is easily accessible, non-invasive, and does not cause adverse effects. When LV EF is >50%, it is considered to be safe to perform HSCT [10]. Further, it is advisable that patients, depending on the risk after HSCT procedures, should undergo a comprehensive clinical and echocardiographic evaluation [8]. In instances where the left ventricular ejection fraction is registered below 40%, it is recommended to start cardioprotective modalities and treatment of heart failure and aim for a therapeutic regimen that minimizes cardiotoxicity. More importantly, when LVEF is >50%, but it is reduced by more than 10% after the therapy starts, alteration of treatment of the main disease or cardioprotective medication should be contemplated [11]. Systolic longitudinal function of the right ventricle (RV) can also be evaluated with the help of echocardiography. The parameter is named S’ and evaluated by tissue Doppler to measure the longitudinal velocity of the tricuspid annulus. S’ has good correlations to radionuclide angiography- and cardiovascular magnetic resonance-determined RV ejection fraction [12].

To our knowledge there is not much published data on the cardiovascular impact of different parts of the HSCT process, in particular, mobilization. In this paper, we aimed to evaluate the impact of the mobilization procedure of autologous HSCT on left and right ventricle sizes and function.

## 2. Materials and Methods

The study was performed prospectively from October 2021 till September 2023 in the Hospital of the Lithuanian University of Health Sciences Kaunas Clinics. The data of 47 patients undergoing autologous HSCT at the Department of Oncology and Hematology were analyzed. Bioethics approvement for the prospective study was obtained (No. BE-2-96). All patients gave their informed consent to take part in the study.

Inclusion criteria were

- Written consent to participate in the study;
- Patients over the age of 18 years scheduled for autologous HSCT for various reasons.

The only exclusion criterion was a patient’s refusal to participate at any time of the study.

Patients underwent autologous transplantation for various reasons. The most common disease was multiple myeloma, following with mantle cell lymphoma, Hodgkin’s lymphoma, diffuse large B cell lymphoma, anaplastic large cell lymphoma, peripheral T cell lymphoma, and Ewing sarcoma. All patients underwent HSC mobilization with chemotherapy and filgrastim 10 µg/kg/d. Multiple myeloma patients had cyclophosphamide 3000 mg/m^2^; mantle cell lymphoma patients had rituximab 375 mg/m^2^ and cytarabine 4 g/m^2^; Hodgkin’s lymphoma patients had cisplatin 30 mg/m^2^ and cytarabine 3.5 g/m^2^; primary central nervous system (PCNS) diffuse large B cell lymphoma patients had cytarabine and thiotepa at different doses and rituximab 375 mg/m^2^; anaplastic large cell lymphoma and peripheral T cell lymphoma patients had a cisplatin-based salvage regimen; and Ewing sarcoma patients followed an IE scheme (ifosfamide with etoposide). All patients underwent apheresis procedures. Peripheral blood stem cells were collected from peripheral blood via an apheresis procedure with a Fresenius Kabi COM.TEC apheresis system. The cells were cryopreserved in a solution of 10% DMSO and autologous plasma. The cryopreservation was performed in a controlled-rate freezer with liquid nitrogen (Consarctic equipment). 

**Figure 1:**
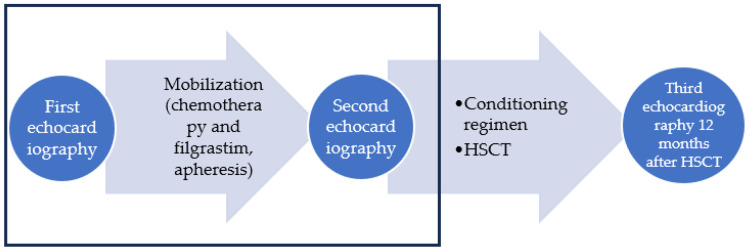
Echocardiography workflow of the study. Rectangular area shows the part of the data analyzed in the article.

During the study period, echocardiography was performed at three time points: first, on evaluation and decision to enroll the patient in the transplantation process (before the mobilization procedure); second, before the transplantation (the conditioning regimen) procedure; and third, at follow-up—12 months after HSCT. In this part of the study, we aimed to evaluate only the impact of mobilization procedure on the heart, so only the measurements of the first two echocardiography examinations were taken into account. The echocardiographic workflow is presented in Figure 1. The data of 47 patients were analyzed. Echocardiography was performed and evaluated by one experienced cardiologist using a Phillips Epiq 7 ultrasound machine. The typical timeframe between the two echocardiography scans was a median (min–max) of 49 (20–168) days. The left ventricular end diastolic diameter (LVEDD) from the parasternal long axis view was measured, and the LVEDD index according to body surface area (BSA) (LVEDDi) was calculated. Left ventricular ejection fraction (LV EF) was calculated from the apical two- and four-chamber views based on left ventricular (LV) volumes, using the modified biplane Simpson method. Volumetric measurements were derived from tracings of the border between the myocardium and LV cavity in the end-systole and end-diastole in the apical two- and four- chamber views. Global LV systolic function was assessed by calculating the difference between the end-diastolic and end-systolic value, divided by the end-diastolic value. Left ventricular global longitudinal strain (GLS) was calculated automatically using a postprocessing system. The endocardial borders were traced in the end-systolic frame of the 2D images from the 3 apical views (two-, three-, and four-chamber). Speckles were tracked frame by frame throughout the LV wall during the cardiac cycle. Segments that failed to track were manually adjusted by the operator. Diastolic LV dysfunction was evaluated using early mitral inflow velocity/mitral annular early diastolic velocity (E/E’) in the apical four-chamber view using pulse and tissue doppler. Right ventricle (RV) end diastolic diameter (RVEDD) was measured at the base from the apical four-chamber view, and tricuspid annular systolic velocity (S’) was also evaluated from the apical four-chamber view using tissue doppler. The measurements were collected before and after the mobilization process (before the conditioning regimen) and compared.

Sub-analysis of the data of multiple myeloma patients was performed. The group was homogenous in the means of chemotherapy treatment—all patients received cyclophosphamide 3000 mg/m^2^ for mobilization.

Patients filled out a survey regarding cardiovascular risk factors (arterial hypertension, smoking, cardiovascular family history, dyslipidaemia, diabetes mellitus). Information about coronary artery disease (CAD) and cardiovascular medication was obtained during the interview and double-checked from medical records. Arterial hypertension was evaluated and graded according to the European Society of Cardiology and European Society of Hypertension guidelines published in 2018. Arterial hypertension was diagnosed if systolic ABP was ≥140 mmHg and/or diastolic was ≥90 mmHg [13]. Cardiovascular family history was defined as cardiovascular event (stroke, myocardial infarction, revascularization procedures) or cardiovascular death in the first-degree relatives (men ≤ 55 year old, women ≤ 65 year old). 

SPSS statistics 20 was used for statistical analysis. Qualitative data are presented as absolute values (N) and percentages (%), and quantitative parameters are given as average ± standard deviation or median. We used Student’s *t*-test, ANOVA, the Mann–Whitney U test, or the Kruskal–Wallis H test to compare quantitative parameters. Chi-square or Fisher’s exact test, as appropriate, was utilized to evaluate categorical variables between groups. The odds ratio (OR) and 95% confidence interval (CI) were used as a measure of the strength of association between cardiovascular risk factors and final RV S’ decrease (<10 cm/s). Association analyses were conducted using univariate logistic regression, followed by multivariate regression. Statistically significant difference was considered when *p* < 0.05.

## 3. Results

Out of 47 patients, there were 27 males (57.4%) and 20 females (42.6%). Median age was 61 (ranging from 18 to 74). A total of 35 (74.5%) patients had multiple myeloma, 4 (8.5%) had mantle cell lymphoma, 3 (6.4%) had Hodgkin’s lymphoma, 2 (4.3%) had PCNS diffuse large B cell lymphoma, 1 (2.1%) had anaplastic large cell lymphoma, 1 (2.1%) had peripheral T cell lymphoma, and 1 (2.1%) had Ewing sarcoma. The values are presented in Table 1.

No patients had clinically relevant signs or symptoms of cardiotoxicity—heart failure, arrhythmias, pericardial effusion, new onset or worsening of arterial hypertension, or acute ischemic syndromes.

Statistically significant difference was observed in the change of RV function (S‘)—it slightly decreased. Mean RV S‘ before mobilization was 13.93 ± 2.85 cm/s and after mobilization 12.19 ± 2.64 cm/s (*p* = 0.003). Six patients had a decrease of S‘ to less than 10 cm/s—a clinically significant loss of RV function (12.8%). Three patients were from the multiple myeloma group and had at least three cardiovascular risk factors. The other three patients had other diseases: one had mantle cell lymphoma, another had Hodgkin’s lymphoma, and the other had diffuse large B cell lymphoma. These patients received either rituximab and cytarabine or cisplatin and cytarabine. Only one of them had no risk factors; the others had two (arterial hypertension and dyslipidaemia) and three (arterial hypertension, diabetes mellitus, and dyslipidaemia). A significant reduction of RV systolic function was observed in 8.6% of patients in the multiple myeloma group and 25% in the other diseases group.

No statistically significant change in LV volumes and systolic and diastolic function and RV size was observed. The values are presented in Table 2. 

Analysis of correlation of cardiovascular risk factors and clinically relevant reduction of S‘ to less than 10 cm/s after mobilization was performed. No statistically significant impact of cardiovascular risk factors was noticed, probably because there were only six patients with clinically significant reduction of S‘. The distribution of risk factors among the patients with and without clinically significant S‘ reduction is presented in Table 3. The results of the univariate logistic regression analysis are presented in Table 4. We could not find a statistically significant impact of cardiovascular risk factors, but the odds ratio shows a tendency for risk increase with CAD, arterial hypertension, and family history of CAD. 

The patients with different cardiovascular risk factors used medication that is considered to be cardioprotective. A total of 17 patients (36.2%) used beta-blockers, 7 patients (14.9%) used angiotensin converting enzyme inhibitors (ACEis), 6 patients (12.8%) used angiotensin receptor blockers (ARBs), and 3 patients (6.4%) used statins.

In the clinically significant S‘ reduction group, three patients were using beta-blockers (50% of all patients with significant S‘ reduction), two patients were using ACEis (33.3%), one was using ARBs (16.7%), and one patient was using statins (16.7%). No statistically significant difference showing cardioprotective properties was observed.

### Sub-Analysis of Patients with Cyclophosphamide-Based Chemotherapy

The data of 35 patients in the multiple myeloma group receiving cyclophosphamide for mobilization were analyzed separately. All values are presented in Table 5. The results were similar. A statistically significant difference was observed in the change of RV systolic longitudinal function (S‘) as well. Mean RV S‘ before mobilization was 13.89 ± 3.12 cm/s, and after mobilization it was 12.20 ± 2.68 cm/s (*p* = 0.018).

In addition, in this subgroup, no statistically significant change in LV volumes, systolic and diastolic function, and RV size was observed. 

Three patients had a clinically significant reduction of S‘ to less than 10 cm/s after mobilization. Due to the very small number of patients, only univariate logistic regression analysis was performed, which did not reveal a statistically significant impact of cardiovascular risk factors.

## 4. Discussion

In this study, we aimed to describe the impact of mobilization procedures in autologous HSCT patients on early cardiovascular toxicity. No clinically relevant signs or symptoms of cardiotoxicity—heart failure, arrhythmias, pericardial effusion, new onset or worsening of arterial hypertension, or acute ischemic syndromes—were observed in all 47 patients. To our knowledge, there are no published data evaluating echocardiographic LV and RV changes during mobilization procedures. We aimed to determine whether there is any significant subclinical impact of the mobilization procedure on the right and left ventricles, not taking into account the difference in the main disease, mobilization chemotherapy regimen, previous cardiotoxic chemotherapy, or cardiovascular risk factors. There is a lot of evidence in the literature that subclinical cardiotoxicity should be evaluated; this helps to find cardiotoxicity in the early phase and avoid further damage, improve outcomes, and reduce the progression of heart failure and other cardiac complications [14,15,16,17,18,19,20,21]. Our results show that there was a statistically significant difference in right ventricular function—there was a slight decrease of S‘ after the mobilization. We found that 12.8% had a significant reduction in RV systolic function—S‘ decreased to less than 10 cm/s. No statistically significant change in RV size, LV size, and systolic and diastolic function was observed. 

S’ is an echocardiographic parameter used for the evaluation of longitudinal RV function. It is evaluated by tissue Doppler to measure the longitudinal velocity of the tricuspid annulus. A number of validation studies have shown good correlations to radionuclide angiography- and cardiovascular magnetic resonance-determined RV ejection fraction [12]. Recent data increasingly indicate that both LV and RV function contribute to clinical heart failure. Functional capacity, quality of life, and overall clinical outcomes are worsened among patients with heart failure when there is evidence of RV dysfunction [22]. Also, RV function is one of the determinants for survival in heart failure, irrespective of the underlying etiology; therefore, it is an important parameter [23]. RV systolic function is affected in shorter periods of time than the left ventricle [24]. Therefore, patients with a clinically significant decrease of RV function should undergo closer follow-up in the further treatment course and start cardioprotective treatment when needed.

Bearing in mind that different chemotherapy regimens might have a different impact on subclinical changes in ventricle size and function, we performed a sub-analysis of the multiple myeloma patient group. All 35 patients had the same chemotherapy regimen—cyclophosphamide 3000 mg/m^2^. RV function in the multiple myeloma subgroup also changed statistically significantly—S‘ slightly decreased. We obtained the same results in the change of RV size, LV size, and systolic and diastolic function—no statistically significant change was observed.

Similar results were noticed by Tekiner et al. [25]. The difference from our study was that they evaluated the whole HSCT process, not only mobilization. They also included patients who had undergone allogeneic HSCT. The study examined 137 patients undergoing autologous and allogeneic HSCT. Echocardiography was performed on the day before and 30 days after HSCT. Changes in S‘ velocity and TAPSE (tricuspid annular plane systolic excursion) also showed a statistically significant decrease, whereas RV and LV sizes and LV EF remained unchanged. This prompts that RV function seems to be affected earlier than LV and can be used to assess early cardiotoxicity in HSCT recipients. Another study supporting this theory and evaluating the effects of cancer chemotherapy showed that RV systolic and diastolic functions were affected in a rather short period of time and earlier than the left ventricle [24].

From all six patients that had a reduction of S‘ to less than 10 cm/s, half of the patients (three) were from multiple myeloma group and had at least three cardiovascular risk factors. The other three patients had other diseases: one had mantle cell lymphoma, another—Hodgkin’s lymphoma, and the other—diffuse large B cell lymphoma. These patients received either rituximab and cytarabine or cisplatin and cytarabine. Only one of them had no risk factors; the others had two (arterial hypertension and dyslipidaemia) and three (arterial hypertension, diabetes mellitus, and dyslipidaemia). The difference of significant reduction in these groups was 8.6% in the myeloma group and 25% in the other diseases group. Although, neither univariate logistic regression nor multivariate regression showed a statistically significant impact of cardiovascular risk factors on RV function, odds ratio showed a tendency toward risk increase with CAD, arterial hypertension, and family history of CAD. These results lead us to believe that multiple risk factors might have an impact on the clinically significant reduction of RV function, but a bigger sample is needed to prove this hypothesis. Also, other chemotherapy regimens used for mobilization and previous treatment might have more impact on right ventricular function than cyclophosphamide and multiple myeloma induction treatment, but further investigation of more cases is needed.

The patients with different cardiovascular risk factors have used medication groups having cardioprotective properties for cardiovascular risk factor correction purposes. These include beta-blockers, ACEis, ARBs, and statins. Our study did not show positive effects on clinically significant reduction of RV systolic function. This should be further investigated with a bigger sample.

According to the literature, cyclophosphamide carries a cardiotoxic risk, especially when used in high doses [26,27,28]. It is an alkylating agent that has both immunosuppressive properties and antineoplastic activity. Therefore, it is used in mobilization regimens, and high-dose cyclophosphamide also has an important role in many conditioning regimens for HSCT [29]. Also, administration of post-transplant high doses of cyclophosphamide in haplo-identical transplant has shown results in preventing graft-versus-host disease [30]. Different types of manifestation and severity of cardiotoxicity have been reported, ranging from pericarditis and arrhythmias to hemorrhagic myocarditis and congestive heart failure [26,28]. Cardiotoxicity is usually observed only after administration of high doses; therefore, dose limitation is very important [31]. The pathophysiology of high-dose cyclophosphamide-associated cardiac toxicity was analyzed in postmortem examinations and is thought to depend on toxic endothelial damage followed by extravasation of toxic metabolites, which results in myocyte damage and interstitial hemorrhage and edema [27]. 

A study conducted by Poreba et al. [32] showed that administration of cyclophosphamide represented independent risk factors for worsening of left ventricular systolic function. In our study, a significant direct impact was noticed on subclinical RV function decrease, but not on LV function. Echocardiography was performed very early, after the mobilization procedure and before starting the conditioning, and this could also support the hypothesis that RV function is an earlier marker of cardiotoxicity than LV function. 

The other agent used for mobilization together with chemotherapy was filgrastim—granulocyte colony stimulating factor (GCSF). There are no available data in the literature about GCSF’s cardiotoxicity. On the contrary, there are a few experimental studies prompting that GCSFs can have regenerative properties for cardiomyocytes. Tomita et al. investigated mice with doxorubicin-induced cardiomyopathy. Their study showed that bone marrow could be one of the sources of regenerated cardiomyocytes in the doxorubicin-induced cardiomyopathic heart. Early administration of GCSF enhanced the migration of bone marrow stem cells into the heart and attenuated doxorubicin-induced cardiotoxicity [33]. Another study tested rats with diabetic cardiomyopathy and found out that GCSF can ameliorate cardiac diastolic dysfunction and morphological damage, leading to fibrosis of the myocardium [34]. There are data that GCSF could reduce carbon monoxide (CO)-induced cardiac ischemia in patients with acute CO poisoning [35]. Moreover, Haybar et al. stated that GCSF can play a role, offering protective chemokines during chemotherapy treatment [36]. These studies show that GCSF might have some cardioprotective properties, but most of them are experimental or very minor and need further investigation.

### Limitations

The main limitation of the study is a relatively small sample. Therefore, the factors influencing clinically significant reduction of RV function could not be defined. Further investigation with a bigger number of patients is needed. The nonhomogeneous group of patients regarding main disease and previous treatment could also be a limitation.

## 5. Conclusions

Mobilization procedure in patients undergoing autologous HSCT are associated with reduced RV systolic function. S‘ could be used as a reliable tool to detect early cardiotoxicity in HSCT patients and guide further follow-up.

## Figures and Tables

**Table 1:** Characteristics of the patients.

**Sex**	
Male, n (%)	27 (57.4)
Female, n (%)	20 (42.6)
**Age**, years (median (min–max))	61 (18–74)
**Main disease**	
Multiple Myeloma, n (%)	35 (74.5)
Mantle cell lymphoma, n (%)	4 (8.5)
Hodgkin‘s lymphoma, n (%)	3 (6.4)
PCNS diffuse large B cell lymphoma, n (%)	2 (4.3)
Anaplastic large cell lymphoma, n (%)	1 (2.1)
Peripheral T cell lymphoma, n (%)	1 (2.1)
Ewing sarcoma, n (%)	1 (2.1)

PCNS: primary central nervous system.

**Table 2:** The change of echocardiographic parameters before and after the mobilization procedure.

Echocardiographic Values	Before Mobilization	After Mobilization	*p*
LVEDD, mm	46.48 ± 4.05	46.02 ± 5.25	0.633
LVEDDi, mm/m^2^	24.85 ± 2.78	24.75 ± 2.93	0.863
LV EF, %	60.49 ± 7.66	59.74 ± 7.08	0.622
GLS, %	−17.45 ± 3.62	−17.59 ± 3.65	0.309
E/E‘	6.58 ± 3.28	6.91 ± 2.65	0.973
RVEDD, mm	36.02 ± 3.83	34.96 ± 4.44	0.217
S‘, cm/s	13.93 ± 2.85	12.19 ± 2.64	0.003

LVEDD: Left ventricular end diastolic diameter; LVEDDi: LVEDD index according to body surface area (BSA); LV EF: left ventricular ejection fraction; GLS: left ventricular global longitudinal strain; E/E’: early mitral inflow velocity/mitral annular early diastolic velocity; RVEDD: right ventricle end-diastolic diameter; S’: tricuspid annular systolic velocity.

**Table 3:** Cardiovascular risk factors among all the patients and risk factors’ distribution among the patients with and without clinically significant S’ reduction after the mobilization procedure.

Cardiovascular Risk Factors	N of Risk Factors among All Patients (%)	Patients with S’ after Mobilization ≥ 10 (n (%))	Patients with S’ after Mobilization < 10 (n (%))	*p*
Coronary artery disease (CAD) ^1^	4 (8.5)	3 (75.0)	1 (25.0)	0.432
Arterial hypertension	23 (48.9)	19 (82.6)	4 (17.4)	0.416
Diabetes mellitus	6 (12.8)	5 (83.3)	1 (16.7)	1.000
Family history of CAD	10 (21.3)	8 (80.0)	2 (20.0)	0.594
Dyslipidaemia	41 (87.2)	36 (87.8)	5 (12.2)	1.000
Smoking	0 (0)	0 (0)	0 (0)	
Previous smoking	7 (14.9)	6 (85.7)	1 (14.3)	1.000

^1^ Previous myocardial infarction or elective stenting.

**Table 4:** Univariate logistic regression analysis showing measure of the strength of association between cardiovascular risk factors and final RV S’ decrease (<10 cm/s).

Cardiovascular Risk Factors	Odds Ratio	95% Confidence Interval	*p*
Coronary artery disease (CAD)	2.533	0.219–29.290	0.457
Arterial hypertension	2.316	0.381–14.079	0.362
Diabetes mellitus	1.440	0.138–14.978	0.760
Family history of CAD	2.062	0.320–13.313	0.447
Dyslipidaemia	0.694	0.067–7.223	0.760
Previous smoking	1.167	0.115–11.814	0.896

**Table 5:** The change of echocardiographic parameters before and after the mobilization procedure in multiple myeloma subgroup patients treated with cyclophosphamide.

Echocardiographic Values	Before Mobilization	After Mobilization	*p*
LVEDD, mm	45.87 ± 3.91	45.08 ± 5.14	0.469
LVEDDi, mm/m^2^	24.49 ± 2.76	24.29 ± 2.90	0.768
LV EF, %	61.67 ± 6.82	60.40 ± 7.09	0.449
GLS, %	−17.87 ± 3.89	−18.00 ± 3.59	0.886
E/E‘	7.39 ± 3.29	7.05 ± 2.77	0.634
RVEDD, mm	36.26 ± 3.97	35.14 ± 4.74	0.290
S‘, cm/s	13.89 ± 3.12	12.20 ± 2.68	0.018

LVEDD: Left ventricular end diastolic diameter; LVEDDi: LVEDD index according to body surface area (BSA); LV EF: left ventricular ejection fraction; GLS: left ventricular global longitudinal strain; E/E’: early mitral inflow velocity/mitral annular early diastolic velocity; RVEDD: right ventricle end diastolic diameter; S’: tricuspid annular systolic velocity.

## Data Availability

The data presented in this study are available on request from the corresponding author.
